# Sarcoidosis-Like Reaction After Chemotherapy Mimicking Metastasis in a Patient With Two Synchronous Tumors: A Case Report

**DOI:** 10.7759/cureus.62939

**Published:** 2024-06-23

**Authors:** Sandra Silva, João Fernandes, Sérgio Campainha, Agostinho Sanches, Cristiana Marques

**Affiliations:** 1 Medical Oncology, Unidade Local de Saúde Gaia/Espinho, Vila Nova de Gaia, PRT; 2 Radiology, Unidade Local de Saúde Gaia/Espinho, Vila Nova de Gaia, PRT; 3 Pulmonology, Unidade Local de Saúde Gaia/Espinho, Vila Nova de Gaia, PRT; 4 Pathology, Unidade Local de Saúde Gaia/Espinho, Vila Nova de Gaia, PRT

**Keywords:** differential histopathological diagnosis, multidisciplinary management, chemotherapy toxicities, drug-induced sarcoidosis-like reaction, gynecological cancer

## Abstract

Sarcoidosis presents a diagnostic challenge due to its diverse clinical manifestations and potential to mimic malignancies. We report a clinical case involving a 46-year-old woman diagnosed with localized synchronous ovarian and endometrial carcinomas treated with surgery. Following adjuvant chemotherapy and radiotherapy, the patient developed suspicious pulmonary micronodules and lymphadenopathy observed in imaging studies, raising concerns about cancer recurrence. Histopathological analysis revealed chronic granulomatous inflammation without evidence of malignancy, leading to a diagnosis of a sarcoidosis-like reaction secondary to chemotherapy. Remarkably, these lesions resolved spontaneously without specific intervention. This case emphasizes the importance of a multidisciplinary approach in managing complex oncological presentations and underscores the significance of histopathological examination in distinguishing between malignancy and chemotherapy-induced sarcoidosis-like reactions.

## Introduction

Sarcoidosis is an idiopathic systemic granulomatous disorder that can present with a variety of clinical manifestations, with the lungs and mediastinal lymph nodes being more commonly affected [1]. Overall, sarcoid reactions occur in 4.4% of carcinomas, 13.8% of patients with Hodgkin's disease, and 7.3% of cases of non-Hodgkin lymphomas [2]. A drug-induced sarcoidosis-like reaction (DISR) refers to a systemic granulomatous syndrome that appears clinically and histopathologically similar to sarcoidosis, distinguishing itself by its temporal relationship with the initiation of an offending drug [3]. The definitive method to differentiate sarcoidosis from DISR is by observing the resolution of DISR after discontinuation of the suspected causative drug [4]. 

In the diagnosis of DISR, as with sarcoidosis, it is essential to thoroughly rule out other potential causes of granulomatous inflammation, such as mycobacterial and fungal infections, as well as sarcoidosis-like reactions associated with malignancy, which typically impact the cancerous organ, its draining lymph nodes, or metastases. To differentiate DISR from cancer-related reactions, immunohistochemical analysis can be utilized, as B lymphocytes and sinus histiocytes are not found in sarcoid granulomas [3-5].

Various drug classes have been linked to DISR, with antiretroviral therapy, tumor necrosis factor-alpha antagonists, interferons, and immune checkpoint inhibitors being the most commonly cited [4]. The occurrence of sarcoidosis during or shortly after chemotherapy is uncommon and has only been documented in a small number of cases involving malignant solid tumors [6]. Regarding specifically carboplatin and paclitaxel chemotherapy, only two cases of DISR have been reported in the literature [7,8]. We report a clinical case where sarcoidosis presents as if it were cancer recurrence, emerging following chemotherapy.

## Case presentation

The patient is a 46-year-old female with a medical history of a myomatous uterus and a significant family history of oncological diseases, including her mother with colon cancer at age 66, her maternal aunt with lung cancer at age 70 (non-smoker), her maternal grandmother with gastric cancer at age 40, her paternal aunt with breast cancer at age 40, and her paternal uncle with melanoma at age 58.

The patient was asymptomatic until March 2019 when she began experiencing fatigue, abdominal pain, and nausea for one month, prompting her visit to the emergency department. During the gynecological examination, a big mass was palpable, occupying all lower quadrants of the abdomen. She was referred for a gynecology consultation and underwent abdominopelvic magnetic resonance imaging, which revealed a potential sarcomatous transformation of uterine fibroids and an apparent area of endometrial thickening. Besides that, an abdominal lesion was observed in the left ovary, measuring around 15cm, and the presence of peritoneal effusion in all quadrants of the abdomen suggested peritoneal metastasis. No lymphadenopathy was identified. In the blood analysis, she presented elevated CA 125 levels of 1170 U/mL (Table 1). She also did a chest CT, which did not reveal any alterations.

**Table 1:** Laboratory results presented in the case report CA 125: Cancer Antigen 125

Parameter	Result	Reference Range
CA 125	1170 U/mL	< 35 U/mL

In June 2019, the patient underwent an exploratory laparotomy during which a total hysterectomy with bilateral salpingo-oophorectomy, infracolic omentectomy, biopsies of parietocolic gutters, appendicectomy, and pelvic and lumbo-aortic lymph node biopsies were performed. At the end of the surgery, there was no evidence of macroscopic residual disease. The anatomopathological examination revealed the presence of two synchronous neoplasms: a mucinous endometrioid carcinoma, G1, of the left ovary, stage IA, and endometrioid carcinoma of the endometrium, G1, with no lymphovascular invasion, dMMR, p53 WT, unknown POLE mutation, as routine screening for it was not recommended at that time, stage III-A, according to 2015 FIGO classification. The CA 125 levels normalized after surgical treatment.

The expression of the mismatch repair proteins, MLH1, MSH2, MSH6, and PMS2, was evaluated by immunohistochemistry, and loss of expression of MLH1 and PMS2 was observed. The patient was referred to a genetics consultation where a multigenic panel, genes BRCA1, BRCA2, BRIP1, RAD51C, RAD51D, MLH1, MSH2, MSH6 e PMS2, was performed, with no pathogenic variants detected. During the multidisciplinary tumor board, surveillance was decided for the ovarian tumor and adjuvant chemoradiotherapy for endometrial cancer.

From September 2019 to February 2020, she underwent three cycles of chemotherapy with carboplatin (6AUC) and paclitaxel (175mg/m^2^) every three weeks. This was followed by radiotherapy - 45 Gy to the entire pelvis, using volumetric modulated arc therapy, with 6 megavolt photons, at 1.8 Gray per day, and brachytherapy - high-dose-rate iridium-192, with three fractions of 5 Gray at 0.5 cm depth, irradiating 3 cm of vaginal mucosa extension, using a 3 cm surface applicator system cylinder. Subsequently, she underwent another three cycles of chemotherapy. The entire treatment proceeded without complications or the need for interruptions, and the patient had good tolerance throughout.

**Figure 1:**
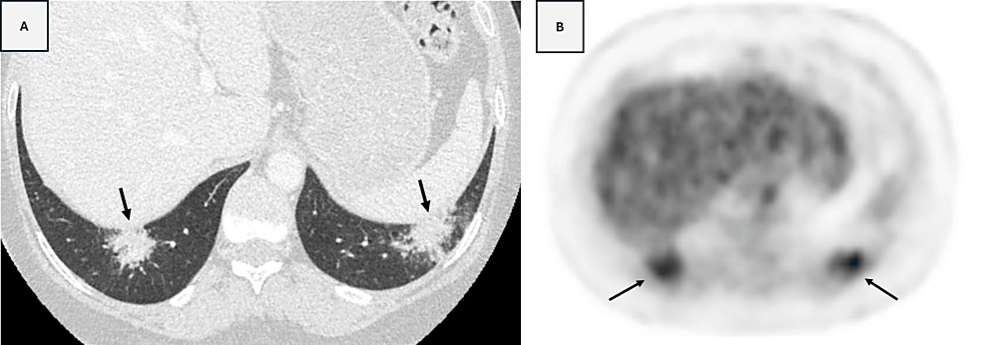
A) CT imaging of the lower lobes displays lung opacities consisting of conglomerates of nodules (arrow), reminiscent of a galaxy (“galaxy sign”), a pattern representing coalescent granulomas commonly associated with pulmonary sarcoidosis. B) PET scan images revealed hypermetabolic pulmonary opacities (arrow) and raised the suspicion of metastatic involvement.

**Figure 2:**
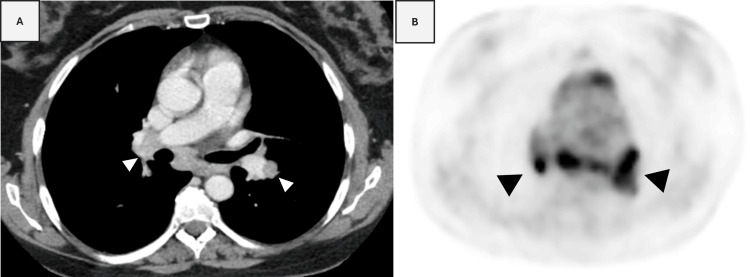
A) CT imaging also showed mediastinal and hilar (arrowhead) enlarged lymph nodes. B). PET scan images confirmed the presence of hypermetabolic mediastinal and hilar lymphadenopathies (arrowhead), which were interpreted as evidence of metastatic disease.

Three months later, in May 2020, during surveillance, the patient remained asymptomatic, CA125 levels stayed negative, but the thoracoabdominopelvic CT scan revealed three pulmonary micronodules. A PET scan performed at this time showed no abnormalities. A repeat CT scan in July 2020 showed dimensional progression of the previous pulmonary nodules, the appearance of a new nodular lesion, and mediastinal and hilar lymphadenopathy. A repeat PET scan raised the possibility of bilateral pulmonary and mediastinal lymph node metastasis (Figures 1, 2). 

The patient remained asymptomatic from a respiratory standpoint, but lesions consistent with erythema nodosum appeared on the lower limbs. Tumor markers remained negative.

**Figure 3:**
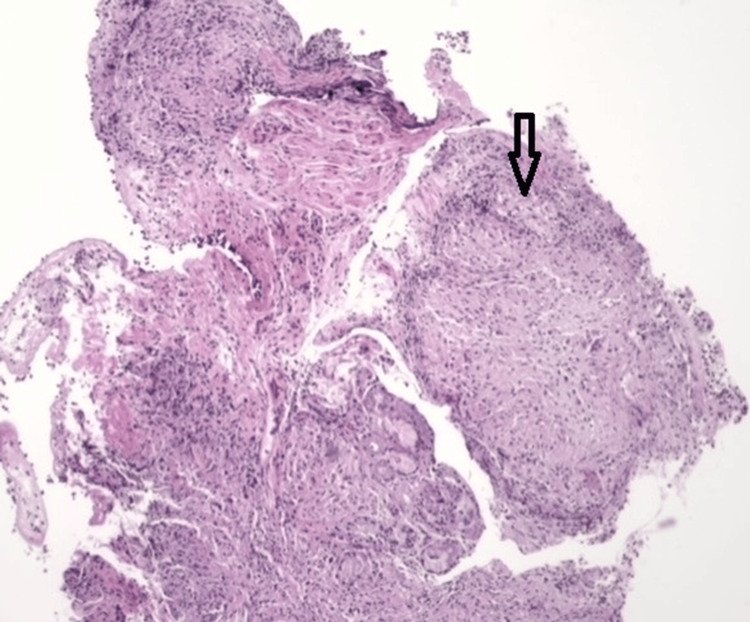
Bronchial mucosa flap with epithelioid granuloma (arrow).

In November 2020, a lung biopsy was performed, and the histopathology report revealed a chronic granulomatous inflammatory process (Figure 3).

**Figure 4:**
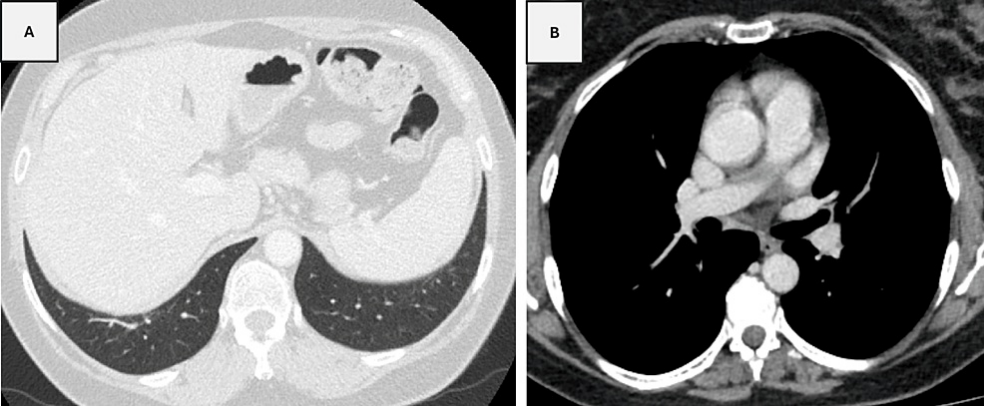
CT imaging showed resolution of the lung opacities (A) and lymphadenopathies (B).

Consequently, the patient was referred to Pulmonology, and in January 2021, she underwent an endobronchial ultrasound bronchoscopy with biopsies of the lymphadenopathies and bronchoalveolar lavage. The histopathology report once again confirmed a granulomatous inflammatory process, with no cells exhibiting morphological characteristics of malignancy. Infectious causes, particularly tuberculosis, were ruled out, as no isolation was observed in microbiological examinations. Lung function was normal and there was no evidence of additional organ involvement. As the patient was asymptomatic, except for erythema nodosum, which was treated with topical corticosteroids, no systemic therapy was proposed. Further CT surveillance documented spontaneous remission of adenopathies and pulmonary lesions (Figure 4).

So, considering the appearance of granulomatous lesions after chemotherapy exposure, their spontaneous resolution upon treatment cessation, and without any directed therapeutic intervention, a sarcoidosis-like reaction secondary to chemotherapy was assumed. The patient currently remains without clinical, laboratory, or imaging evidence of recurrence.

## Discussion

Development of sarcoidosis during or immediately after chemotherapy is rare since most of the reported drugs have been antiretroviral therapy, tumor necrosis factor-α antagonists, interferons, and immune checkpoint inhibitors [4]. There are only two previous reports on the development of sarcoidosis associated with a combination of chemotherapy paclitaxel and carboplatin [7,8]. In 2019, a review of the World Health Organization pharmacovigilance database analyzed 2425 cases of DISR and identified significant correlations with immunotherapy, including drugs like pembrolizumab, nivolumab, and ipilimumab (n = 103), BRAF and MEK inhibitors like dabrafenib, vemurafenib, trametinib, and cobimetinib (n = 37), and other drugs such as tumor necrosis factor-alpha inhibitors, interferon, and PEG-interferon [9]. Therefore, this clinical case stands out for documenting a rare event secondary to chemotherapy, specifically with this regimen that is so commonly used in the treatment of gynecological tumors.

The immunopathogenesis of sarcoidosis is not well understood, so it is unclear whether a DISR is due to a drug triggering sarcoidosis, increasing the immune system's vulnerability to sarcoidosis, worsening an asymptomatic case of sarcoidosis, or resulting in a condition separate from sarcoidosis. However, the most commonly proposed pathophysiological mechanism is that the drug amplifies an abnormal immune response, leading to the formation of granulomas [3].

DISR and sarcoidosis present the same clinical, biological, radiological, and pathological features, so distinguishing between the two relies primarily on observing DISR resolution after discontinuation of the suspected drug and its recurrence upon rechallenge [3,4]. However, re-administration of the suspected drug is usually avoided, often leaving the diagnosis based on temporal patterns. In this specific case report, the resolution of the pulmonary nodules and adenopathies identified on the chest CT scan was observed after the permanent discontinuation of chemotherapy, without the need for any treatment directed at sarcoidosis, such as systemic corticosteroids. This made it possible to establish a cause-and-effect relationship between the chemotherapy treatment and the imaging and pathological findings, leading to a diagnosis of DISR.

Another entity distinct from DISR is a sarcoid-like reaction to malignancy, which is essentially a sarcoid reaction occurring in or around the tumor or in regional lymph nodes [5]. In this clinical case, the patient did not present any imaging abnormalities in the lung parenchyma or mediastinal lymph nodes at the time of diagnosis; they only appeared after the treatment directed at the oncological disease. Furthermore, the sites affected by granulomas are not the most common sites of metastasis in endometrial and ovarian cancer. Therefore, all the previously described facts point toward the diagnosis of DISR.

This patient had the initial diagnosis of two synchronous tumors, one primary in the ovary and the other primary in the endometrium, confirmed upon pathological review of the surgical specimen. So, the initial hypothesis was to consider it as a recurrence of one of the neoplasms. The biopsy was crucial in allowing the diagnosis and preventing the wrong decision to assume a recurrence of the oncological disease and initiate incorrect treatment. At present, distinguishing between sarcoidosis and neoplasia still relies solely on histological examination. Although there are promising advancements, such as new radiotracers like 18F-FLT and novel acquisition techniques like dual time point PET CT, these innovations have not yet become standard in clinical practice [10].

## Conclusions

We report a case of DISR to chemotherapy directed at gynecologic cancer treatment, notable for the rarity of described cases of chemotherapy-induced sarcoidosis, particularly associated with the combination of carboplatin and paclitaxel. These two cytotoxic agents, or possibly just one of them, may have induced the reaction; however, further studies will be required to explain the pathogenesis of sarcoidosis during or after chemotherapy.

This clinical case also highlights that the radiological findings, including those from the PET scan, may not be able to distinguish between cancer recurrence and sarcoidosis. Therefore, it is crucial to conduct a careful study of the imaging, ensuring good integration with the patient's clinical history, and, if possible, always confirming the presence of malignancy histologically.
